# Identification of Essential Genes Associated With Prodigiosin Production in *Serratia marcescens* FZSF02

**DOI:** 10.3389/fmicb.2021.705853

**Published:** 2021-07-22

**Authors:** Xianbo Jia, Fangchen Liu, Ke Zhao, Junjie Lin, Yu Fang, Shouping Cai, Chenqiang Lin, Hui Zhang, Longjun Chen, Jichen Chen

**Affiliations:** ^1^Institute of Soil and Fertilizer, Fujian Academy of Agricultural and Sciences, Fuzhou, China; ^2^College of Resources and Environment, Fujian Agriculture and Forestry University, Fuzhou, China; ^3^Faculty of Life Sciences, Fujian Agriculture and Forestry University, Fuzhou, China; ^4^Institute of Forest Protection, Fujian Academy of Forestry Sciences, Fuzhou, China

**Keywords:** Ez-Tn5 transposon, *Serratia marcescens*, prodigiosin, essential genes, regulation

## Abstract

Prodigiosin is a promising secondary metabolite produced mainly by *Serratia* strains. To study the global regulatory mechanism of prodigiosin biosynthesis, a mutagenesis library containing 23,000 mutant clones was constructed with the EZ-Tn5 transposon, and 114 clones in the library showed altered prodigiosin production ability. For 37 of the 114 clones, transposon insertion occurred on the prodigiosin biosynthetic cluster genes; transposon inserted genes of the 77 clones belonged to 33 different outside prodigiosin biosynthetic cluster genes. These 33 genes can be divided into transcription-regulating genes, membrane protein-encoding genes, and metabolism enzyme-encoding genes. Most of the genes were newly reported to be involved in prodigiosin production. Transcriptional levels of the *pigA* gene were significantly downregulated in 22 mutants with different inserted genes, which was in accordance with the phenotype of decreased prodigiosin production. Functional confirmation of the mutant genes involved in the pyrimidine nucleotide biosynthesis pathway was carried out by adding orotate and uridylate (UMP) into the medium. Gene complementation confirmed the regulatory function of the EnvZ/OmpR two-component regulatory system genes *envZ* and *ompR* in prodigiosin production.

## Introduction

Prodigiosin is a pigment produced by strains of *Serratia* spp., *Vibrio* spp. (Borić et al., 2011), *Streptomyces coelicolor* (Liu et al., 2017), and other bacterial species. Prodigiosin has generated great interest in recent years due to its broad biological activities such as anticancer and antimicrobial properties (Bérdy, 2005). Research has mainly focused on three aspects: the optimization of the isolation and the production of prodigiosin-producing strains (Yip et al., 2019), the biological activity of prodigiosin (Li et al., 2018), and the synthesis and regulation mechanisms of prodigiosin in bacterial strains (Pan et al., 2019; Sun et al., 2020). The synthesis and regulation mechanism of prodigiosin are essential for its effective production and modification and the foundation for its further applications.

Genes in *Serratia* spp. and *Streptomyces* spp. have been identified, and these genes form a gene cluster in the genome (Williamson et al., 2006). In *Serratia* spp., the prodigiosin synthesis gene cluster arranged in the following order: *pigA*, *pigB*, *pigC*, *pigD*, *pigE*, *pigF*, *pigG*, *pigH*, *pigI*, *pigJ*, *pigK*, *pigL*, *pigM*, and *pigN*. Some of the synthesis genes are involved in 2-methyl-3-n-amyl-pyrrole (MAP) synthesis, some are involved in the production of 4-methoxy-2,2'-bipyrrole-5-carbaldehyde (MBC), and some are involved in the terminal step of prodigiosin synthesis with MAP and MBC (Williamson et al., 2006). Besides these 14 genes, *Serratia* sp. ATCC 39006 consisted of a gene *pigO* in its prodigiosin synthesis gene cluster, which was different with many *Serratia* spp. strains (Williamson et al., 2006).

Effective synthesis of prodigiosin is regulated by many genes in *Serratia* spp. Genes of the quorum-sensing and two-component regulatory systems are two main kinds of genes that were reported to play roles in regulating prodigiosin synthesis. SmaI/SmaR (Thomson et al., 2000; Slater et al., 2003; Fineran et al., 2005a) and SpnI/SpnR (Horng et al., 2002) are two types of quorum-sensing systems that have been reported to control prodigiosin production in *Serratia* spp. strains. PigQ/PigW (Fineran et al., 2005a), PhoB/PhoR (Gristwood et al., 2009), RssB/RssA (Horng et al., 2010), and EepR/EepS (Stella et al., 2015) are four kinds of two-component regulatory systems that can regulate the synthesis of prodigiosin. In addition, there are also many other transcription factors involved in prodigiosin synthesis including the positive regulators PigP (Fineran et al., 2005a), PigT (Fineran et al., 2005b), PigS (Gristwood et al., 2011), PigR, and PigV (Fineran et al., 2005a) and the negative regulators PigX (Fineran et al., 2007) and HexS (Tanikawa et al., 2006). Previous studies on the regulatory genes were mainly focused on *Serratia* sp. strain ATCC 39006 (Ravindran et al., 2019), other prodigiosin biosynthetic regulating genes in *Serratia* spp. were to be discovered. With Tn5 transposition system, a total of 365 genes that affect the prodigiosin synthesis levels of *S. coelicolor* were identified, including 17 genes in the prodigiosin biosynthetic gene cluster and many regulating genes (Xu et al., 2016), which showed Tn5 transposition system was a powerful tool for identifying genes involved in prodigiosin biosynthesis.

*Serratia marcescens* FZSF02 was previously isolated from soil and demonstrated to be a very promising strain for prodigiosin production (Lin et al., 2019). In this study, a mutant library was constructed to identify the regulatory genes for prodigiosin synthesis in this strain to enhance prodigiosin production through a genetic approach.

## Materials and Methods

### Plasmids, Primers, Strains, and Culture Conditions

The plasmids and strains used in this study are listed in Table 1. The primers used in this study are listed in Table 2. *Serratia marcescens* FZSF02 (GenBank accession number of the whole genome: CP053286.1) is a prodigiosin-producing strain that was used to construct the Tn5 mutant library. *Escherichia coli* DH5α was used to construct complementary plasmids of *ompR* and *envZ*. *Serratia marcescens* FZSF02 and the mutants were cultured with modified Luria-Bertani (LB) medium (tryptone (10 g/L), yeast extract (5 g/L), and NaCl (5 g/L)). *Escherichia coli* DH5α was cultured with Luria-Bertani (LB) medium. Antibiotics were used when necessary at the following concentrations: kanamycin (100 mg/L), ampicillin (100 mg/L), and tetracycline (50 mg/L).

**Table 1:** Plasmids and strains used in this study.

Plasmid or strain	Description^a^	References or sources
**Plasmids**		
pMD19T	Cloning plasmid, Amp^r^	Takara
pMD19-*pigA-*kan	Plasmid pMD19T containing kanamycin resistance gene and *pigA*, Amp^r^, Km^r^	This work
pRK415	Broad-host-range, low-copy-number expression vector, Tet^r^	Keen et al., 1988
pRK415-envZ	pRK415 containing envZ, Tet^r^	This work
pRK415-ompR	pRK415 containing ompR, Tet^r^	This work
**Strains**		
*Serratia marcescens*		
FZSF02	Wild-type strain	This work
WT(pRK415)	Wild-type strain FZSF02 transformed with plasmid pRK415, Tet^r^	This work
envZ::Tn5	Wild-type strain FZSF02 with *envZ* inserted by Tn5 transposon, Kan^r^	This work
ompR::Tn5	Wild-type strain FZSF02 with ompR inserted by Tn5 transposon, Kan^r^	This work
envZ::Tn5(pRK415-envZ)	Mutant strain *envZ*::Tn5 transformed with plasmid pRK415-*envZ*, Km^r^, Tet^r^	This work
ompR::Tn5(pRK415-ompR)	Mutant strain *ompR*::Tn5 transformed with plasmid pRK415-*ompR*, Km^r^, Tet^r^	This work
△envZ	Wild-type strain FZSF02 with inframe deletion of *envZ* gene, Km^r^	This work
△ompR	Wild-type strain FZSF02 with inframe deletion of *ompR* gene, Km^r^	This work
*Escherichia coli*		
DH5α	Cloning host	Laboratory collection

a Km^r^, Amp^r^, and Tet^r^ indicate resistance to kanamycin, ampicillin, and tetracycline, respectively.

**Table 2:** Primers used in this study.

Primer name	Sequence (5'-3')	Description	References or sources
LAD1	ACGATGGACTCCAGAG(G/C/A)N(G/C/A)NNNGGAA	For High Tail-PCR	Liu and Chen, 2007
LAD3	ACGATGGACTCCAGAG(T/A/C)N(A/G/C)NNNCCAC	For High Tail-PCR	Liu and Chen, 2007
AC1	ACGATGGACTCCAGAG	For High Tail-PCR	Liu and Chen, 2007
F389	TCAAGCATTTTATCCGTACTCCTG	For High Tail-PCR	This study
F536	CGGTTGCATTCGATTCCTGTTTGTA	For High Tail-PCR	This study
F772	TAGGTTGTATTGATGTTGGACGAG	For High Tail-PCR	This study
16SF	CGTTACTCGCAGAAGAAGCA	For qPCR	This study
16SR	TCACCGCTACACCTGGAA	For qPCR	This study
PigAF	CGCCATCTTCCACGATTCAA	For qPCR	This study
PigAR	CATTAGCCGACACTGTTCCA	For qPCR	This study
KanF	CGTTGCCAATGATGTTACAGATG	For qPCR	This study
KanR	AATGCTTGATGGTCGGAAGAG	For qPCR	This study
PromotF	TCTCAACCATCATCGATGAATT	For complementary expression of *envZ* and *ompR*	This study
PromotR	AACACCCCTTGTATTACTGT	For complementary expression of *envZ* and *ompR*	This study
EnvZF	ACAGTAATACAAGGGGTGTTATGAGGCGATTGCGCTTTTCA	For complementary expression of *envZ*	This study
EnvZR	TCAGGCGTTTTCCCTGGCCGTT	For complementary expression of *envZ*	This study
OmpRF	ACAGTAATACAAGGGGTGTTATGCAAGAGAATCATAAGATC	For complementary expression of *ompR*	This study
OmpRR	TCATGCCTTGCTGCCGTCCGGT	For complementary expression of *ompR*	This study
OmpRFF	ATGCAAGAGAATCATAAGATCCTG	For gene knockout of *ompR*	This study
OmpRFR	CATCGATGATGGTTGAGAGTCGGCGCCGATTTCCAGCCCCA	For gene knockout of *ompR*	This study
OmpRBF	CTCGATGAGTTTTTCTAAGGCAAATTCAAACTGAACCTCGGC	For gene knockout of *ompR*	This study
OmpRBR	TCATGCCTTGCTGCCGTCCGGTAC	For gene knockout of *ompR*	This study
EnvZFF	ATGCAAGAGAATCATAAGATC	For gene knockout of *envZ*	This study
EnvZFR	CATCGATGATGGTTGAGATCATGCCTTGCTGCCGTCCGGT	For gene knockout of *envZ*	This study
EnvZBF	CTCGATGAGTTTTTCTAAAGATGGCGTCGGGCGTCAAGCAGC	For gene knockout of *envZ*	This study
EnvZBR	TCTCCATCGGCAACGGAATATACG	For gene knockout of *envZ*	This study
KF	TCTCAACCATCATCGATGAATTGT	For gene knockout	This study
KR	TTAGAAAAACTCATCGAGCATCAA	For gene knockout	This study

### Mutagenesis Library Construction

Competent cells for electroporation were prepared using the following method. The *S. marcescens* FZSF02 strain was cultured with 50 ml of LB medium in a flask (250 ml) at 37°C and 200 rpm. When the OD_600_ value reached approximately 1.0, the flask with the broth was incubated in an ice-water mixture for 30 min. Then, the broth was centrifuged in a 50-ml centrifuge tube at 5,000 rpm and 4°C for 10 min. After that, the supernatant was poured carefully, and the bacterial precipitate was resuspended in precooled sterilized ddH_2_O (0°C) and centrifuged at 5,000 rpm and 4°C for 10 min. These washing and centrifuging steps were repeated twice. The precipitate was then resuspended carefully with precooled 10% (v/v) glycerol and centrifuged at 5,000 rpm and 4°C for 10 min, and this was repeated twice. The bacterial precipitate was resuspended in 0.5 ml of precooled 10% glycerol and stored at −80°C with 100 μl in a 1.5-ml tube.

The EZ-Tn5™ <KAN-2>Tnp Transposome™ Kit (TSM99K2, Epicentre) was used to construct the mutagenesis library. Electronic pulse was operated with 0.1-cm electroporation cuvettes (Cat: 1652083, Bio-Rad) on the Gene Pulser Xcell™ (Bio-Rad) with the following electroporation parameters: 25 μF, 200 ohm, and 1800 V. After electronic pulse, the broth was immediately transferred into 1 ml of LB medium and incubated at 37°C and 200 rpm for 1 h. The incubated broth was diluted properly and screened on LB agar plates containing kanamycin (100 mg/L). Plates were incubated at 28°C until the color of the clones became red. Clones showing white or redder colors were selected and reserved for further research.

### Copy Number of the Tn5 Transposon of the Mutant Strains

The copy number of the Tn5 transposon of the mutant strains was calculated with the Ct shift method (Kolacsek et al., 2017). In detail, *PigA* was chosen as the reference gene because there was only one copy of the *pigA* gene in the genome of *S. marcescens* FZSF02, and the Tn5 transposon copy number was calculated by assaying the copy number of the kanamycin resistance gene. A standard plasmid pMD19-*pigA*-*kam* containing both *pigA* and *kam* was constructed with pMD-19 T (Takara). Then, two qPCR standard curves were obtained using the primers (Table 2) of *pigA* and *kam* separately with the plasmid as the template. The Ct shift of the two curves at every point is a constant value. If there was only one Tn5 copy in the mutant strain genome, the Ct shift value obtained from the qPCR performed with the mutant strain genomic DNA as the template would be the same as the Ct shift value for the standard curves.

### Identification of the Transposon Insertion Sites of the Mutants

High-tail PCR was used to identify the transposon insertion sites of the mutants (Liu and Chen, 2007; Jia et al., 2017). The primers used in this method are shown in Table 2.

### Details of Plasmid and Strain Construction

The broad host range plasmid pRK415 was used to express the genes in mutant strains for genetic complementation. For expression, a promoter sequence (5'-TCTCAACCATCATCGATGAATTGTGTCTCAAAATCTCTGATGTTACATTGCACAAGATAAAAATATATCATCATGAACAATAAAACTGTCTGCTTACATAAACAGTAATACAAGGGGTGTT-3') was amplified with the primers PromotF and PromotR (Table 2). The *ompR* and *envZ* genes were amplified by PCR with the OmpRF/OmpRR and EnvZF/EnvZR primers. Overlapping PCR was used for the fusion of gene sequences with promoters. The PCR products were digested with the restriction endonucleases EcoR1 and HindIII and then ligated with pRK415, which was also digested with the restriction endonucleases EcoR1 and HindIII. The recombinant plasmids pRK415-*envZ* and pRK415-*ompR* were amplified in *E. coli* DH5α separately and then transformed into the mutant strains *envZ*::Tn5 and *ompR*::Tn5 separately by the electronic pulse method.

The homologous recombination method was used to inactivate genes. Briefly, the forward homologous sequence of about 300 bp was amplified with primers OmpRFF and OmpRFR, the backward homologous sequence of about 300 bp was amplified with primers OmpRBF and OmpRBR; kanamycin resistant gene was amplified with primers KF and KR; these three sequences were spliced by Overlap-PCR. The PCR products were transformed into *S. marcescens* FZSF02 for the homologous recombination deletion of *ompR*. Positive clones were screened with LB agar plates with kanamycin firstly and confirmed with PCR method. The gene *envZ* was also partially deleted with the same procedure.

### Measurement of Prodigiosin Production and Bacterial Growth

Prodigiosin was extracted as follows. The fermentation broth was properly diluted with acidified methanol (4 ml of 1 mol/L HCl and 96 ml of methanol) and shocked vigorously; after 30 min of standing, the mixture was centrifuged at 10,000 × *g* for 10 min, and the supernatant was removed for prodigiosin quantification. Prodigiosin production can be evaluated by the absorbance value at 535 nm. The growth conditions of the strains can be assayed by comparing the *OD*_600_ values of the fermentation broth of the strains.

### Quantitative Real-Time Polymerase Chain Reaction

Quantitative real-time polymerase chain reaction (qRT-PCR) was used to evaluate the expression levels of *pigA* in the mutants. To obtain the total RNA of the mutants, the strains were incubated in 50 ml LB liquid medium at 28°C and 180 rpm for 18 h. Total RNA of each strain was extracted with an RNA extraction kit (DP430; Tiangen, Beijing, China); a PrimeScript™ RT reagent kit with the gDNA Eraser (RR047; TaKaRa, Dalian, China) was used to prepare cDNA with total RNA. 16S rDNA was chosen as the reference gene with the 16SF and 16SR primers (Table 2). The expression levels of *pigA* were chosen to reflect the expression levels of genes of the whole prodigiosin biosynthesis cluster with the pigAF and pigAR primers (Table 2). qRT-PCR was performed with SuperReal PreMix Plus (SYBR Green; Tiangen, Beijing, China) on a QuantStudio 6 Real-Time PCR System (Applied Biosystems) using the following primers: 16S rDNA, 16SF, and 16SR; pigA, pigAF, and pigAR (Table 2). Gene expression levels of mutant strains were determined using the 2^-ΔΔCT^ method with the relative fold-difference expressed against the wild-type strain.

## Results

### EZ-Tn5-Mediated Mutation Library Construction of *S. marcescens* FZSF02

We constructed two mutation libraries with the EZ-Tn5 transposon and obtained approximately 23,000 mutant clones. The probability that the transposon inserted one certain gene was calculated with the formula *P* = 1 − (1 − *X*/*G*)^n^, where *P* is the property that one gene was found to be inserted by the transposon, *X* is the average length of the genes of the studied strain (1,800 bp for the *S. marcescens* FZSF02 strain used in this study), *G* is the genome length of the studied strain (5.6 × 10^6^ bp for the *S. marcescens* FZSF02 strain), and *n* is the number of mutant clones obtained in the mutation library (Krysan et al., 1999; Song et al., 2015a). We calculated that the property one certain gene was inserted was above 99% in this study.

We tested 10 mutants with the Ct shift method and confirmed that each mutant had one copy of the Tn5 transposon in the genome (Supplementary Figure S4 and Supplementary Table S3 in Supplementary Material).

### Mutants With Altered Prodigiosin Production Ability in the Library

A total of 114 clones showed altered prodigiosin-producing ability. Thirty-seven mutants were found in the prodigiosin biosynthetic cluster genes, which are *pigA*, *pigB*, *pigC*, *pigD*, *pigE*, *pigF*, *pigH*, *pigI*, *pigL*, and *pigM*. The mutant number and the transposon insertion sites of each mutant are shown in Supplementary Table S1 in Supplementary Material. All the mutants with the prodigiosin biosynthetic cluster genes inserted by the Tn5 transposon lost prodigiosin-producing ability.

Seventy-seven mutants (Supplementary Table S2 in Supplementary Material) showed altered prodigiosin-producing ability, which involved 33 outside prodigiosin biosynthetic cluster genes (Table 3). Fourteen mutants were found to have the transposon insertion of the Tn5 transposon in the gene encoding dihydroorotate dehydrogenase (quinone), seven mutants had the gene encoding the type VI secretion protein, and four clones had the genes encoding CpxA, osmolarity response regulator/DNA-binding response regulator OmpR, transcriptional regulator SlyA, long-chain fatty acid outer membrane, and DEAD/DEAH box helicase. For other genes, the number of mutants was 1 to 3. The name of mutants and the transposon insertion sites of these genes are shown in the Supplementary Table S2 in Supplementary Material.

**Table 3:** Characterization of the Tn5 mutants of *Serratia marcescens* FZSF02 with altered prodigiosin production.

Strain number	Gene function	Protein accession number	RED fold change	OD_600_	OD_600_ fold change	RED fold change/OD_600_ fold change
A22	4-Hydroxy-tetrahydrodipicolinate reductase	AXX26100.1	1.050 ± 0.055^a^	0.63 ± 0.003	0.3	3.5
A7	DEAD/DEAH box helicase	QEV96117.1	1.128 ± 0.065	2.18 ± 0.039	1.038	1.09
C22	Bifunctional hydroxy-methylpyrimidine kinase/ hydroxy-phosphomethylpyrimidine kinase	AXX23263.1	1.068 ± 0.069	2.16 ± 0.022	1.029	1.04
WT			1	2.10 ± 0.007	1	1
G4	Polysaccharide export protein Wza	AIA49532.1	0.923 ± 0.038	2.01 ± 0.011	0.957	0.96
D18	DNA helicase IV	AXX25120.1	0.819 ± 0.056	1.89 ± 0.021	0.9	0.91
A9	67 bp at 5' side: aspartate-semialdehyde dehydrogenase64 bp at 3' side: antibiotic transporter		0.671 ± 0.034	1.81 ± 0.007	0.862	0.78
E7	Long-chain-fatty-acid--CoA ligase	AXX24125.1	0.759 ± 0.130	2.07 ± 0.011	0.986	0.77
E8	Long-chain fatty acid outer membrane	AXX23453.1	0.732 ± 0.038	2.05 ± 0.011	0.976	0.75
A15	Undecaprenyl phosphate-alpha-L-ara4N exporter	AXX24660.1	0.716 ± 0.046	2.12 ± 0.021	1.01	0.71
G5	Succinylglutamate desuccinylase	AXX24046.1	0.567 ± 0.038	1.84 ± 0.006	0.876	0.65
B9	Lipopolysaccharide assembly protein B (LapB)	AXX24235.1	0.284 ± 0.004	1.65 ± 0.003	0.786	0.36
E6	N5-carboxyaminoimidazole ribonucleotide mutase	AXX25705.1	0.118 ± 0.003	1.7 ± 0.029	0.81	0.15
E21	Protein TolR	ASM10958.1	0.113 ± 0.077	1.65 ± 0.013	0.786	0.14
G6	Peptidoglycan-associated lipoprotein	QHJ25464.1	0.095 ± 0.007	1.63 ± 0.014	0.776	0.12
D19	ATPase and specificity subunit of ClpA-ClpP ATP-dependent serine protease	AXX25189.1	0.104 ± 0.009	2.03 ± 0.042	0.967	0.11
F8	RNA polymerase sigma factor RpoS	AXX25992.1	0.079 ± 0.061	2.0 ± 0.018	0.952	0.08
E13	Transcriptional regulatory protein RcsA	AXX23844.1	0.076 ± 0.006	1.99 ± 0.017	0.948	0.08
E35	Pitrilysin	AXX27124.1	0.069 ± 0.004	1.77 ± 0.019	0.843	0.08
C6	Outer membrane channel protein TolC	AIA46858.1	0.022 ± 0.003	1.76 ± 0.008	0.838	0.03
E12	Sensory histidine kinase in two-component regulatory system with OmpR(EnvZ)	AXX26987.1	0.019 ± 0.002	2.0 ± 0.018	0.952	0.02
F22	Type VI secretion protein	QIR64882.1	0.018 ± 0.004	1.99 ± 0.017	0.948	0.02
A19	Succinate dehydrogenase flavoprotein subunit	AXX25611.1	0.010 ± 0.002	1.17 ± 0.006	0.557	0.02
C32	Orotidine 5'-phosphate decarboxylase	AYU90687.1	0.007 ± 0.002	0.61 ± 0.009	0.290	0.02
E23	Envelope stress sensor histidine kinase CpxA	AXX20343.1	0.014 ± 0.005	2.07 ± 0.011	0.986	0.01
F19	Multidrug efflux system protein AcrB	AGE16869.1	0.004 ± 0.002	2.02 ± 0.019	0.962	0.004
A4	Transcriptional regulator SlyA	AXX24606.1	0.004 ± 0.003	1.98 ± 0.017	0.943	0.004
C30	Dihydroorotase	AXX24967.1	0.001	0.75 ± 0.008	0.357	0.003
F20	Aspartate carbamoyltransferase	AXX26282.1	0.001	0.70 ± 0.01	0.333	0.003
D6	Osmolarity response regulator/DNA-binding response regulator OmpR	AXX26986.1	0.001	1.90 ± 0.021	0.905	0.001
E37	Pyridoxamine 5'-phosphate oxidase	ALL40291.1	0	1.33 ± 0.024	0.633	0
F11	Dihydroorotate dehydrogenase (quinone)	QHJ25929.1	0	0.54 ± 0.003	0.257	0
D2	F0F1 ATP synthase subunit B	AXX20431.1	0	0.23 ± 0.01	0.11	0
B7	Carbamoyl-phosphate synthase large subunit	AXX26098.1	0	0.22 ± 0.008	0.1	0

a The values represent the means and standard errors.

To test the effects of transposon insertion, prodigiosin producing ability and growth ability of 33 strains that represented 33 outside prodigiosin biosynthetic cluster genes were assayed. After incubation in liquid LB medium at 28°C for 48 h, prodigiosin production as OD_535 nm_ value and the growth of culture as OD_600 nm_ of each mutant strain were assayed. The OD_535 nm_ value and OD_600_ value ratio of the mutant strains to the wild-type strain reflected the prodigiosin-producing ability and the growth conditions of the mutant strains, respectively. The ratio of the prodigiosin fold change to the OD_600_ fold change in each mutant strain reflected prodigiosin production per unit biomass of this strain (Table 3). Among these outside prodigiosin biosynthetic cluster genes, the transposon insertion of three genes encoding the DEAD/DEAH box helicase, bifunctional hydroxy-methylpyrimidine kinase/hydroxy-phosphomethylpyrimidine kinase, and 4-hydroxy-tetrahydrodipicolinate reductase led to an increase in prodigiosin production. Mutant strains with other genes inserted by transposon showed decreased prodigiosin production to varying degrees (Table 3). Some mutants showed a lower prodigiosin-producing ability than the wild-type strain by more than 5-fold, but the effects on growth were much less than the prodigiosin-producing ability. These mutants involved genes encoding sensor protein CpxA (AXX26828.1), ATPase and specificity subunit of ClpA-ClpP ATP-dependent serine protease (AXX25189.1), multidrug efflux system protein AcrB (AGE16869.1), sensory histidine kinase EnvZ in two-component regulatory system (AXX26987.1), RNA polymerase sigma factor RpoS (AXX25992.1), type VI secretion protein (QIR64882.1), transcriptional regulatory protein RcsA (AXX23844.1), transcriptional regulator SlyA (AXX24606.1), osmolarity response regulator/DNA-binding response regulator OmpR (AXX26986.1), pitrilysin (AXX27124.1), outer membrane channel protein TolC (AIA46858.1), N5-carboxyaminoimidazole ribonucleotide mutase (AXX25705.1), protein TolR (ASM10958.1), and peptidoglycan-associated lipoprotein (QHJ25464.1). Some mutants showed a much lower prodigiosin-producing ability along with a significantly decreased growth ability, which involved genes encoding pyridoxamine 5'-phosphate oxidase (ALL40291.1), succinate dehydrogenase (SDH) flavoprotein subunit (AXX25611.1), dihydroorotase (AXX24967.1), aspartate carbamoyltransferase (AXX26282.1), orotidine 5'-phosphate decarboxylase (AYU90687.1), dihydroorotate dehydrogenase (quinone) (QHJ25929.1), F0F1 ATP synthase subunit B (AXX20431.1), and carbamoyl-phosphate synthase large subunit (AXX26098.1).

**Figure 1:**
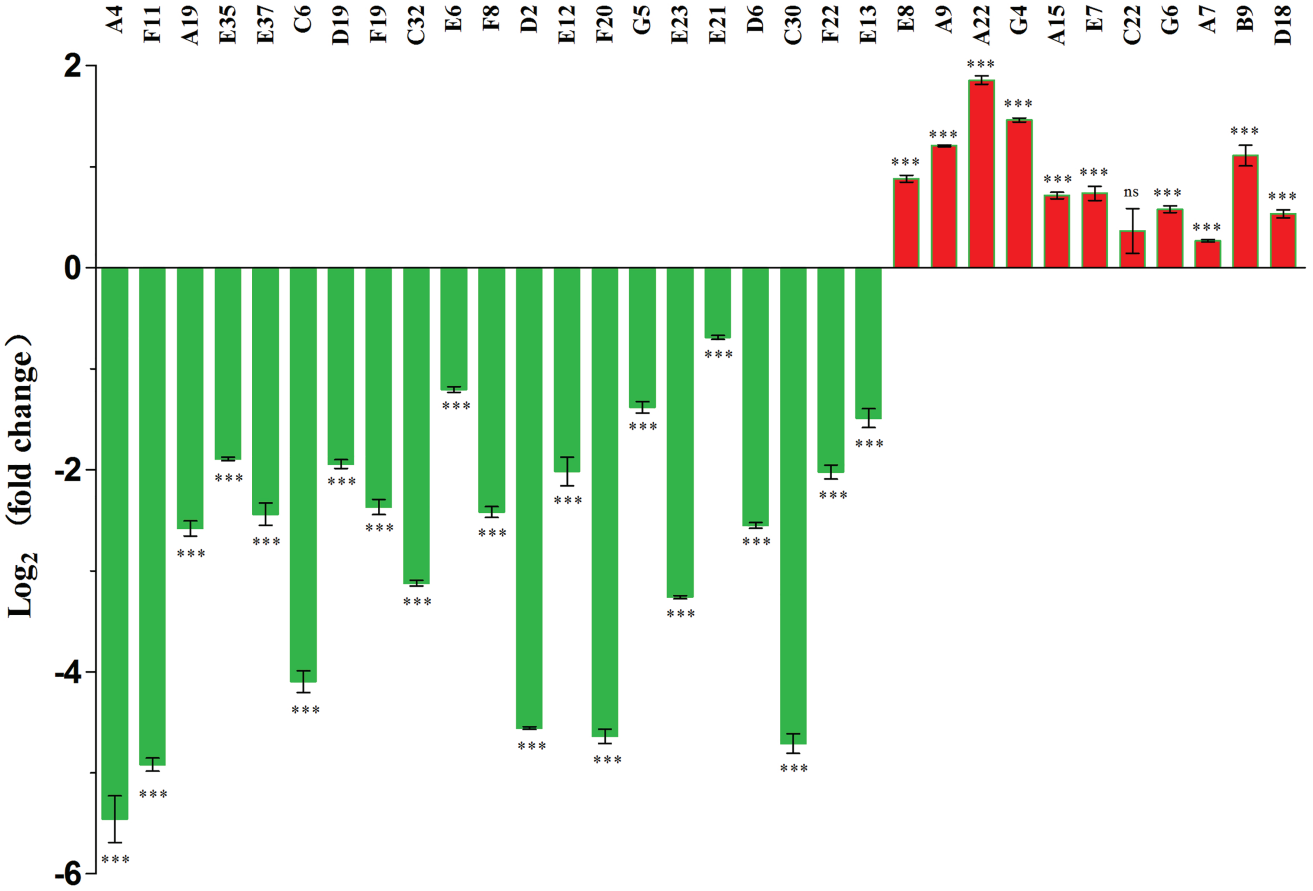
Evaluation of the *pigA* expression level in 32 mutant strains with different genes inserted by the Tn5 transposon. Numbers from A4 to D18 in the above figure represent the mutants chosen for qPCR. Means ± SDs from three independent experiments are shown. Significant differences by unpaired *t*-test between the mutant strains and wild type strain are shown as follows: ***p < 0.001, and ns, no significance.

Transcriptional levels of the *pigA* gene in 32 mutants were assayed with qPCR. Every selected mutant had an outside prodigiosin biosynthetic cluster gene that was different from other mutants. The transcriptional level of *pigA* can reflect the transcriptional level of the whole prodigiosin biosynthetic cluster. Transcriptional levels of *pigA* in 22 mutants were significantly downregulated (Figure 1), and all these mutants showed a much lower prodigiosin-producing ability (Table 3). Mutants A7, C22, and A22 with enhanced prodigiosin production ability showed upregulated *pigA* transcriptional levels (Figure 1), indicating they might negatively regulate prodigiosin in this strain.

### Functional Confirmation of the Mutant Genes

**Figure 2:**
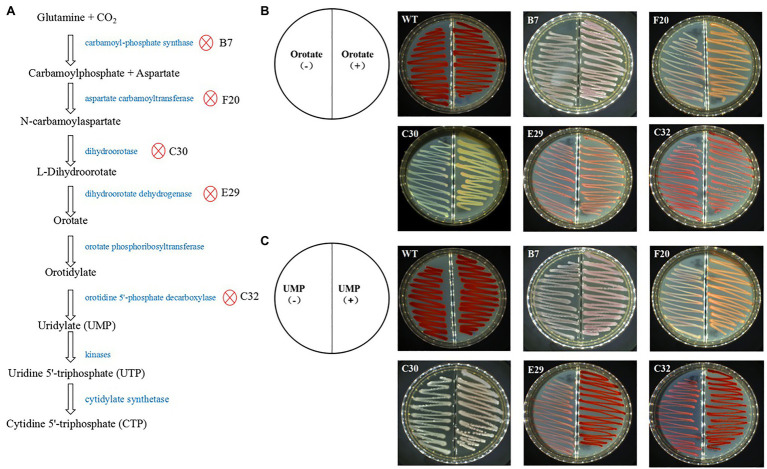
Functional certification of prodigiosin production in the mutant genes involved in pyrimidine nucleotide biosynthesis. **(A)** Proteins of the pyrimidine nucleotide biosynthesis pathway and the proteins, in which the encoding genes were inserted by the Tn5 transposon. **(B)** Rescue effect on prodigiosin production by adding orotate into the medium. The prodigiosin-producing ability can be partially rescued for B7, F20, C30, and E29 but not for C32. **(C)** The rescue effect on the prodigiosin production of UMP for the mutant strains. Prodigiosin producing ability can be partially rescued for B7, F20, C30, E29, and C32. The concentration of orotate and UMP was 0.1 g/L; the incubation conditions of the strains were 28°C for 36 h.

Mutants with pyrimidine nucleotide biosynthesis pathway genes showed a significantly decreased production of prodigiosin. Of these mutants, the transposon insertion of four genes encoding carbamoyl-phosphate synthase, aspartate carbamoyltransferase, dihydroorotase, and dihydroorotate dehydrogenase blocks the biosynthesis of orotate and uridylate (UMP); transposon insertion of a gene encoding orotidine 5'-phosphate decarboxylase will lead to biosynthesis blockade of UMP only (Figure 2A). When orotate or UMP was added to the LB agar plates, mutant B7 with the inserted gene encoding carbamoyl-phosphate synthase, F20 with the inserted gene encoding aspartate carbamoyltransferase, C30 with the inserted gene encoding dihydroorotase, and E29 with the inserted gene encoding dihydroorotate dehydrogenase showed improved prodigiosin-producing ability to a certain extent compared with the treatments with no orotate or UMP addition (Figures 2B,C). Mutant strain C32 with the inserted gene encoding orotidine 5'-phosphate decarboxylase showed significantly enhanced prodigiosin-producing ability when UMP was added to the medium but no enhanced effects was observed when orotate was added (Figures 2B,C). These results demonstrated that these inserted genes are essential for *S. marcescens* FZSF02 in the production of prodigiosin.

**Figure 3:**
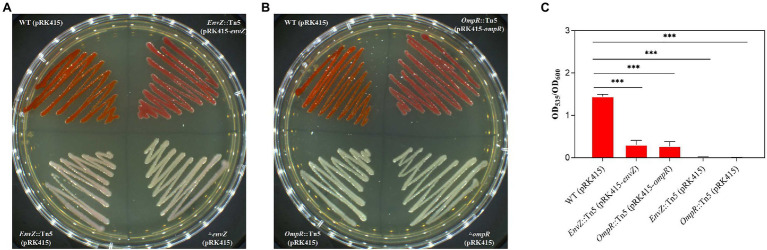
Functional certification of EnvZ and OmpR on prodigiosin-producing ability by gene knockout and complementation. **(A)** The complementary strain *EnvZ*::Tn5 (pRK415-*envZ*) was constructed by transferring the pRK415-*envZ* plasmid with a tetracycline-resistant gene into the mutant *envZ*::Tn5 strain. The wild-type (WT) strain, Tn5-inserted *envZ*::Tn5 mutant and gene knockout *ΔenvZ* strain were transfected with pRK415 to confer tetracycline resistance and used as controls. **(B)** The complementary *ompR*::Tn5 (pRK415-*ompR*) was constructed by transferring pRK415-ompR into the *ompR*::Tn5 strain. The other three strains were similar to those described in **(A)**. **(C)** Prodigiosin producing ability of WT (pRK415) strain, complementary strain *envZ*::Tn5 (pRK415-*envZ*), complementary strain *ompR*::Tn5 (pRK415-*ompR*), mutant strain *envZ*::Tn5 (pRK415), and *ompR*::Tn5 (pRK415). Means ± SDs from three independent experiments are shown. Significant differences in prodigiosin producing ability relative to the related strains/WT by unpaired *t*-test are shown as follows: ***p < 0.001. All the strains were incubated on LB agar with tetracycline (50 mg/L) at 28°C for 36 h.

Transposon insertions of *envZ* and *ompR*, two genes of the EnvZ/OmpR two-component regulatory system, led to the loss of the prodigiosin-producing ability for mutants E12, D6, F12, E26, and D9. The in-frame deletion of either *envZ* or *ompR* led to loss of the prodigiosin-producing ability (Figures 3A,B). When gene complementation was carried out by transforming pRK415-*envZ* in mutant E12 and pRK415-*ompR* in strain D6, the prodigiosin-producing abilities were restored to a certain extent (about 21.4% for E12 and 18.6% for D6 compared with WT strain; Figures 3A–C).

## Discussion

In this study, we constructed a transposon mutant library with the EZ-Tn5™ <KAN-2>Tnp Transposome™ Kit and obtained 114 clones that showed altered prodigiosin-producing ability involving prodigiosin biosynthetic cluster genes and outside prodigiosin biosynthetic cluster genes. Prodigiosin biosynthetic cluster genes identified in this study were very similar to other reported *S. marcescens* strains either on the nucleotide sequences or on the gene orders (GenBank accession number of the whole genome of FZSF02: CP053286.1). However, most of the genes that play regulatory roles in prodigiosin biosynthesis in this study have not been reported earlier. There were two types of prodigiosin biosynthetic clusters in *Serratia* spp., the *Serratia* 39006 type and the *Serratia* 274 type (Harris et al., 2004; Williamson et al., 2006); prodigiosin biosynthetic cluster of *S. marcescens* FZSF02 belonged to the *Serratia* 274 type (Supplementary Figure S1 in Supplementary Material), but promoter sequences of FZSF02 prodigiosin biosynthetic cluster showed some difference from that of *Serratia* sp. ATCC 274 (Supplementary Figures S2, S3 in Supplementary Material). There are fewer studies on genes that regulate prodigiosin biosynthesis of *Serratia* 274 strains than of *Serratia* 39006 strains, and only *luxS* (Coulthurst et al., 2004), *rpoS* (Qin et al., 2020), *slyA* (Pan et al., 2020), and *cpxA* (Sun et al., 2020) have been previously reported.

Two-component regulatory systems played an important role in regulating prodigiosin biosynthesis. In this study, *cpxA* of the CpxA/CpxR two-component regulatory system and *envZ* and *ompR* of the EnvZ/OmpR two-component regulatory system were two-component regulatory system genes, and the transposon insertion of these three genes with the Tn5 transposon led to significantly decreased prodigiosin production (72.7-fold for *cpxA*, 54-fold for *envZ* and 839.3-fold for *ompR*). It was confirmed that in *S. marcescens* JNB 5-1, CpxR negatively regulated the transcription of the cluster by binding to the promoter of the prodigiosin cluster, and CpxA negatively regulated the expression of *cpxR*; therefore, the *cpxA* mutant showed decreased prodigiosin production (Sun et al., 2020). Significantly decreased prodigiosin production was also found in the CpxA transposon insertion mutant, the regulatory mechanism of which might be similar to that of the *S. marcescens* JNB 5-1. *EnvZ* and *ompR* positively regulated prodigiosin production, as confirmed by both the transcription levels (Figure 1) and production levels (Figure 3; Table 3). The EnvZ/OmpR two-component regulatory system was reported to regulate various properties of bacteria, such as phase variation, osmotic tolerance, and motility virulence (Tipton and Rather, 2017), but there are no reports about their relationship with prodigiosin biosynthesis. *EnvZ* and *ompR* are two newly confirmed genes that regulate prodigiosin biosynthesis, and the regulatory mechanisms are still unknown.

In addition to two-component regulatory system genes, some other transcription-regulating genes were also found to regulate prodigiosin production in this study, including *rcsA*, which encodes the transcriptional regulatory protein RcsA, *slyA*, which encodes the transcriptional regulator SlyA, and *rpoS*, which encodes the RNA polymerase sigma factor RpoS. Transposon insertions of *rcsA*, *slyA*, and *rpos* showed decreased prodigiosin production (13.1-, 246.2-, and 12.6-fold, respectively) compared with that of the wild-type strain, and the *pigA* transcriptional levels of the three mutants were all downregulated to varying degrees. RpoS was reported to positively regulate prodigiosin production in *S. marcescens* 1912768R and negatively regulate prodigiosin production in *Serratia* sp. ATCC 39006 (Wilf and Salmond, 2012; Qin et al., 2020). SlyA has been confirmed to positively regulate prodigiosin biosynthesis in *S. marcescens* JNB5-1, and the transcription of *slyA* itself was negatively controlled by MetR (Pan et al., 2020). More details on the regulation of prodigiosin by *slyA* are still unknown. RcsA was a positive regulatory gene of prodigiosin production in this study, which was accordance with the recent study in *S. marcescens* JNB5-1 (Pan et al., 2021).

Some mutants with genes encoding membrane proteins inserted showed significantly decreased prodigiosin production. These genes included *acrB*, which encodes the multidrug efflux system protein AcrB, *tolC*, which encodes the outer membrane channel protein TolC, *tolR*, which encodes the protein TolR, *pal*, which encodes the peptidoglycan-associated lipoprotein, *tai4*, which encodes the type VI secretion protein Tai4, and *lapB*, which encodes lipopolysaccharide assembly protein B (LapB). Transposon insertions of *acrB* and *tolC*, which encode two membrane channel proteins, both showed significantly decreased prodigiosin production (249.5- and 45.9-fold, respectively). The AcrAB-TolC efflux pump system is well-known for its role in the multidrug resistance of bacteria (Abdel-Halim et al., 2019; Nolivos et al., 2019). However, no clues can be obtained to explain the potential regulatory mechanism of these two genes for prodigiosin production from current studies. *TolR* and *pal* are two genes of the Tol-Pal system, and the transposon insertion of *tolR* and *pal* showed 8.8- and 10.6-fold decreased prodigiosin production, respectively. The Tol-Pal system plays important roles in cell division and phospholipid transport from the membrane of bacteria (Szczepaniak et al., 2020). It was found that different kinds of oils can significantly enhance prodigiosin production of many strains in the *Serratia* genus (Wei and Chen, 2005; Abdul Manas et al., 2020), including the *S. marcescens* FZSF02 strain, which was used in this study (Lin et al., 2019). The transposon insertions of *tolR* and *pal* may obstruct liquid usability for prodigiosin biosynthesis. The transposon insertion of *lapB* resulted in decreased prodigiosin production (3.5-fold). LapB coordinates the assembly of proteins involved in LPS synthesis at the plasma membrane in *E. coli* (Klein et al., 2014), but its function in *S. marcescens,* especially in prodigiosin biosynthesis, needs to be studied further. The transposon insertion of *tai4* decreased prodigiosin (54.8-fold). Tai4 (a type VI secretion protein) is a compound of the type VI secretion system (T6SS). T6SS is a novel multi-subunit needle-like secretion apparatus and plays multiple roles in processes of bacterial life cycles such as interspecies competition, biofilm formation, and virulence-related processes (Zhang et al., 2013). The *tai4* gene was also a newly found gene involved in prodigiosin production in this study.

Some genes encoding metabolic enzymes and other enzymes were also found to affect prodigiosin biosynthesis. The transposon insertion of genes encoding dihydroorotase, aspartate carbamoyltransferase, orotidine 5'-phosphate decarboxylase, dihydroorotate dehydrogenase (quinone), and carbamoyl-phosphate synthase large subunit led to a decrease in prodigiosin by more than 100-fold. These five genes are involved in cytidine 5'-triphosphate (CTP) biosynthesis, and CTP or its metabolic intermediates may be the material for prodigiosin biosynthesis. When *atpF*, which encodes F0F1 ATP synthase subunit B, was inserted, the mutant strain lost the prodigiosin-producing ability and showed decreased growth ability. Mutating *atpF* led to a decrease in ATP supply, and prodigiosin biosynthesis might be an ATP consumption process. This can also be demonstrated to a certain extent by an earlier study showing that ATP enhanced prodigiosin production in *S. marcescens* (Haddix et al., 2008). The transposon insertion of *ptrA*, which encodes pitrilysin, and *clpA*, which encodes ClpA-ClpP ATP-dependent serine protease chaperone ClpA, both led to decreased prodigiosin production (14.5-fold and 9.6-fold, respectively) of the strains. Pitrilysin (EC 3.4.24.55) is a kind of metalloendopeptidase; pitrilysin can cleave the -Tyr16−/−Leu-, -Phe25−/−Tyr-bonds of the oxidized insulin B chain and acts on other substrates of less than 7 kDa such as insulin and glucagon (Finch et al., 1986; Ding et al., 1992; Anastasi et al., 1993). *ptrA* is located between *recB* and *recC* on the genomes of both *E. coli* (such as the K12 strain) and *S. marcescens* FZSF02, which was used in this study; however, its biological functions, including how it regulates prodigiosin biosynthesis in bacteria, are still unclear., ClpA is a part of the ClpA-ClpP ATP-dependent serine protease complex (Hoskins et al., 2001), which plays an important role in refolding and degrading proteins, an essential process for the viability and growth of cells (Song et al., 2015b). Lipopeptide biosynthesis in *Pseudomonas fluorescens* is regulated by ClpAP *via* the pathway-specific LuxR-type regulator MassAR, the heat shock proteins DnaK and DnaJ, and proteins of the TCA cycle (Brugarolas et al., 2011). ClpA may also indirectly regulate prodigiosin of *S. marcescens* FZSF02 by regulating other unknown pathways. The N5-carboxyaminoimidazole ribonucleotide mutase encoded by the *purE* gene is a key enzyme for the biosynthesis of inosine monophosphate (IMP), the common precursor of AMP and GMP (Brugarolas et al., 2011). The transposon insertion of *purE* may cause a decreased supply of purines and ATP; the lack of purines and ATP may lead to decreased prodigiosin production (8.5-fold decrease in this study) and biomass. Pyridoxamine 5'-phosphate oxidase encoded by the gene *pdxH* participates in the salvage pathway of pyridoxal 5'-phosphate, an important coenzyme that participates in many metabolic reactions. The transposon insertion of *pdxH* of *S. marcescens* FZSF02 showed decreased growth ability (OD_600_ value of 1.33) compared with that of the wild-type strain (OD_600_ value of 2.1; Table 3), which was in accordance with the previous finding that *pdxH* mutants of *E. coli* showed poorer growth (Lam and Winkler, 1992; Ito and Downs, 2020). Meanwhile, the transposon insertion of *pdxH* resulted in the loss of the prodigiosin-producing ability for *S. marcescens* FZSF02, but the mechanism of which remains unknown. *SdhA* encodes the SDH flavoprotein subunit, a key subunit of SDH that converts succinate to fumarate during the tricarboxylic acid cycle. SdhE is necessary for the incorporation of FAD into SdhA in *S. marcescens* (McNeil et al., 2012) and the mutation of *sdhE* showed impaired growth (McNeil et al., 2012) and 50% reduction in prodigiosin (McNeil et al., 2012). The transposon insertion of *sdhA* of *S. marcescens* FZSF02 in this study led to loss of the prodigiosin production ability and impaired growth (OD600 value 1.17 vs. 2.1 of WT) (Table 3). Perhaps SdhE indirectly affects prodigiosin production by regulating the activity of SdhA; the regulatory details of SdhA on prodigiosin biosynthesis also need to be further studied.

In this study, we constructed a mutation library with strain *S. marcescens* FZSF02 harboring the 274-type prodigiosin cluster. We identified 33 genes involved in the regulation of prodigiosin production, 29 of which were first reported in this study. This study identified potential regulatory genes at the genome level. These genes will provide a better understanding of the regulatory details of the secondary metabolite prodigiosin; they might also be valuable targets of metabolism engineering for producing prodigiosin and other metabolites.

## Data Availability Statement

The datasets presented in this study can be found in online repositories. The names of the repository/repositories and accession number(s) can be found in the article/Supplementary Material.

## Author Contributions

XJ and JC designed the study and wrote the manuscript. XJ, FL, KZ, JL, YF, SC, LC, HZ, and CL performed the experiments. XJ, FL, and JC analyzed the results. All authors contributed to the article and approved the submitted version.

### Conflict of Interest

The authors declare that the research was conducted in the absence of any commercial or financial relationships that could be construed as a potential conflict of interest.
