# Supplementary material for: Identification of Essential Genes Associated With Prodigiosin Production in Serratia marcescens FZSF02
Source: Front Microbiol. 2021 Jul 22;12:705853. doi: 10.3389/fmicb.2021.705853 (PMC8339205; doi:10.3389/fmicb.2021.705853)
Supplement: Supplementary file 3 [file Data_Sheet_3.DOCX]

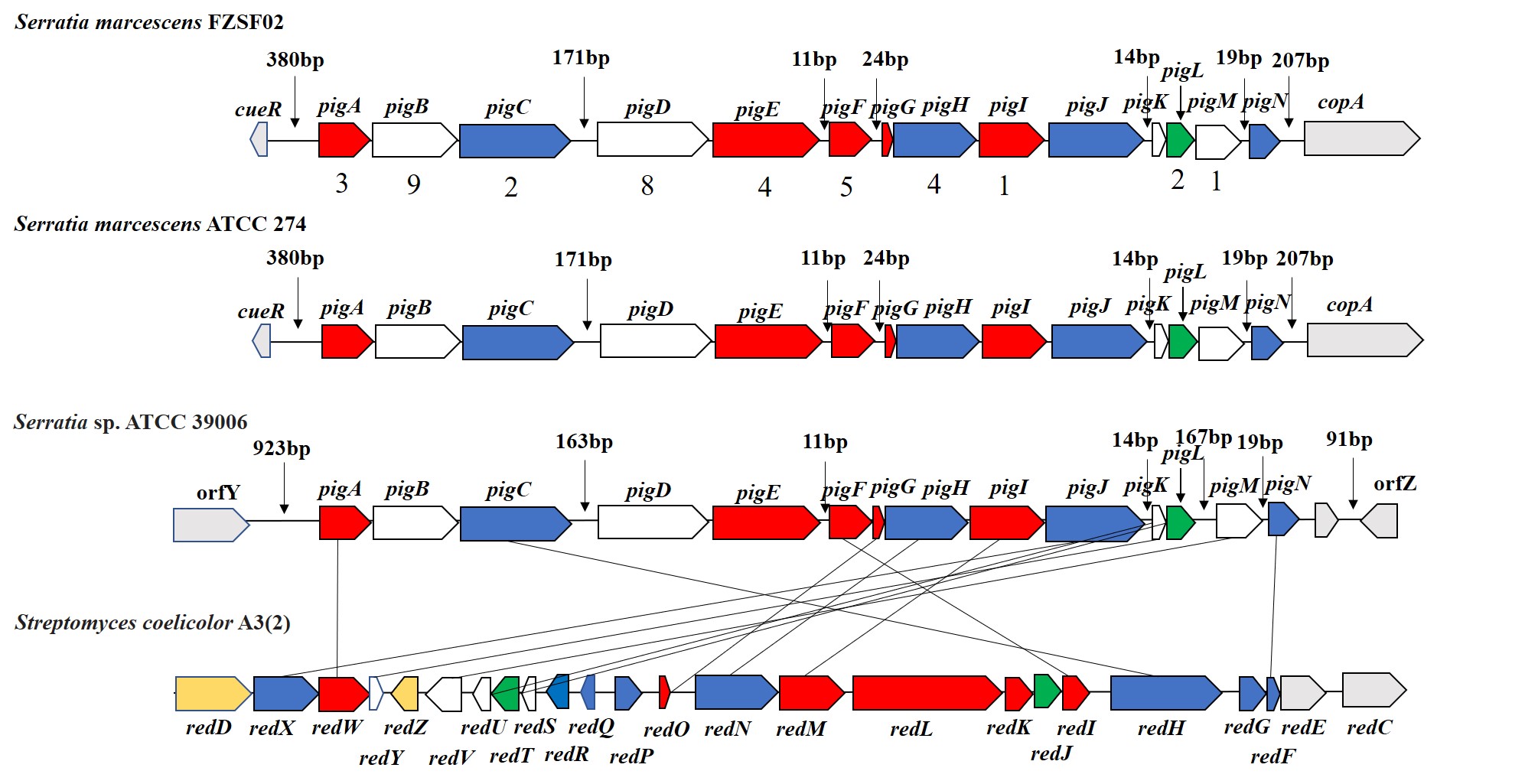


**Figure S1** Prodigiosin synthesis gene cluster of different bacteria strains. Numbers below the prodigiosin cluster of FZSF02 represent the mutant numbers of the corresponding genes.


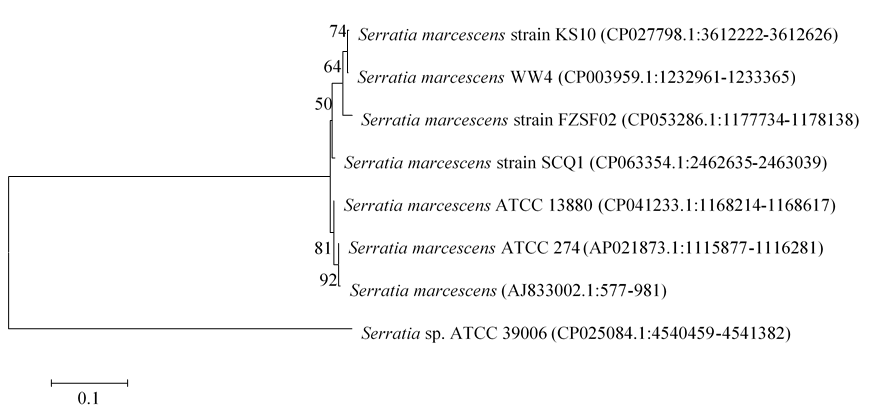


**Figure S2** Phylogenetic tree based on promoter sequences of pig clusters from different *Serratia* strains. Maximum Likelihood method was used and the Bootstrap Replications was 500. Pig cluster promoter sequences of *Serratia* strains divided into two types; promoter of FZSF02 in this study belongs the ATCC274 type.

**
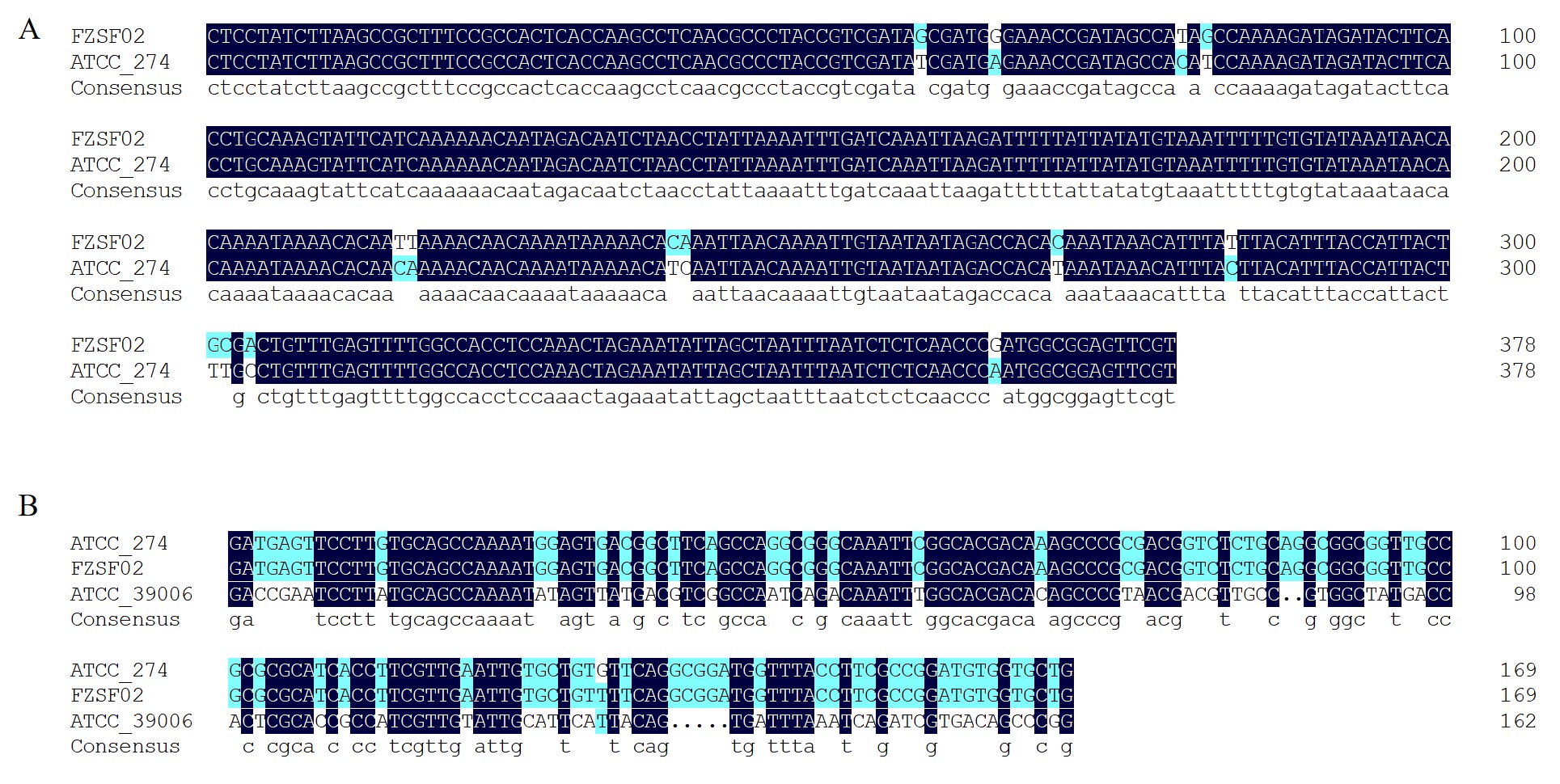
Figure S3** Multiple alignment of pig promoter sequences, long ITS sequences between *pigC* and *pigD*. (A) Multiple alignment of promoter sequences of pig clusters between FZSF02 and ATCC 274. (B) Multiple alignment of long ITS sequences between *pigC* and *pigD* of different strains.

**
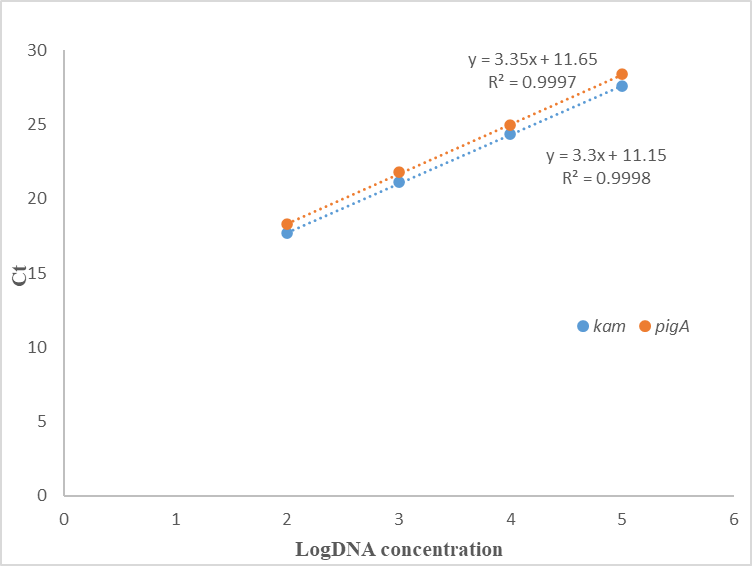
**

**Figure S4** Standard curves regression of *pigA* and *kam* with pMD19-pigA-kan.

| **Strain** | **Ct ( *pigA)-* Ct ( *kam)*** |
| --- | --- |
| A22 | 0.61 |
| A7 | 0.4 |
| C22 | 0.52 |
| G4 | 0.52 |
| D18 | 0.7 |
| A9 | o.44 |
| E7 | 0.46 |
| E8 | 0.57 |
| A15 | 0.47 |
| G5 | 0.77 |

**Table S3** Ct ( *pigA)-* Ct ( *kam)* values of different mutants
